# Hexosamine pathway but not interstitial changes mediates glucotoxicity in pancreatic β-cells as assessed by cytosolic Ca^2+^ response to glucose

**DOI:** 10.18632/aging.100647

**Published:** 2014-03-29

**Authors:** Kazuhiro Yanagida, Yuko Maejima, Putra Santoso, Zesemdorj Otgon-Uul, Yifei Yang, Kazuya Sakuma, Kenju Shimomura, Toshihiko Yada

**Affiliations:** ^1^2nd Department of Internal Medicine, Kagoshima University School of Medicine, Kagoshima, Japan; ^2^ Department of Physiology, Division of Integrative Physiology, Jichi Medical University, School of Medicine, Shimotsuke, Tochigi, Japan

**Keywords:** Glucotoxicity, hexosamine pathway, insulin secretion

## Abstract

Hyperglycemia impairs insulin secretion as well as insulin action, being recognized as the glucotoxicity that accelerates diabetes. However, the mechanism underlying the glucotoxicity in pancreatic β-cells is not thoroughly understood. Hyperglycemia alters glucose metabolism within β-cells and interstitial conditions around β-cells, including elevated osmolarity and increased concentrations of insulin and ATP released from overstimulated β-cells. In this study, to explore direct effects of these alterations on β-cells, single β-cells isolated from rat islets were cultured for 3 days with high (22.3 mM) glucose (HG), compared with control 5.6 mM glucose, followed by their functional assessment by measuring cytosolic Ca^2+^ concentration ([Ca^2+^]_i_). The [Ca^2+^]_i_ response to a physiological rise in glucose concentration to 8.3 mM was impaired in b-cells following culture with HG for 3 days, while it was preserved in β-cells following culture with non-metabolizable L-glucose and with elevated osmolarity, insulin and ATP. This HG-induced impairment of [Ca^2+^]_i_ response to 8.3 mM glucose was prevented by adding azaserine, a hexosamine pathway inhibitor, into HG culture. Conversely, culture with glucosamine, which increases the hexosamine pathway flux, impaired [Ca^2+^]_i_ response to 8.3 mM glucose, mimicking HG. These results suggest that the HG-associated abnormal glucose metabolism through hexosamine pathway, but not elevated osmolarity, insulin and ATP, plays a major role in the glucotoxicity to impair the secretory function of pancreatic β-cells.

## INTRODUCTION

Type 2 diabetes is caused by insulin resistance (IR) and/or impaired insulin secretion. In the process of progression to type 2 diabetes, IR often takes place as the initial event. IR, even at pre-diabetic state, is a risk factor for neuropathy [1] and nephropathy [2], for macrovascular disease [3], and for death and morbidity [4]. IR causes impaired glucose tolerance, a state with a larger and/or longer postprandial elevation of blood glucose. The elevated blood glucose impairs various organs, being recognized as glucotoxicity [5,6], which is linked to both diabetic complications and progression of aging [4]. The glucotoxicity that takes place in pancreatic islet β-cells results in reduction of insulin secretion and overt type 2 diabetes. However, the factor that links hyperglycaemia to dysfunction of pancreatic β-cells is not thoroughly understood.

**Figure 1:**
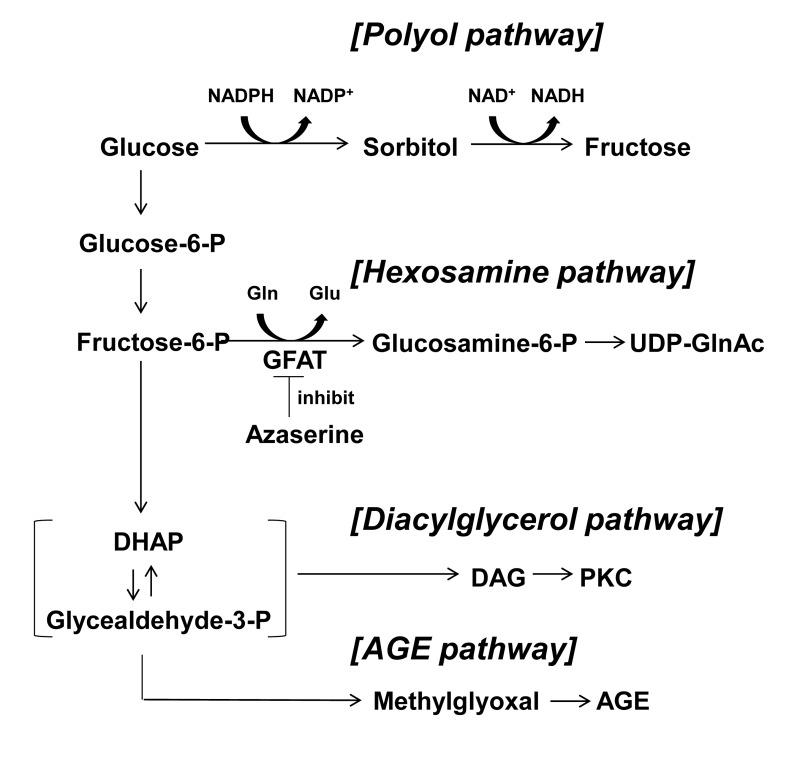
Four major pathways of glucose metabolism underlying glucotoxicity. Azaserine inhibits GFAT. GFAT; glutamine: fructose-6-phosphate amidotransferase, DHAP: dihydroxyacetone phosphate, DAG: diacylglycerol, AGE: advanced glycation product.

Hyperglycemia alters glucose metabolism within β-cells and elevates osmolarity around β-cells. It is also possible that elevated glucose over-stimulates the release of insulin and ATP form β-cells, resulting in increased interstitial concentrations of insulin and ATP. Regarding the abnormal glucose metabolism associated with glucotoxicity, four glucose-driven or glycolysis metabolites-deriven pathways have been reported (Fig. 1) [7,8]. Among these, hexosamine pathway has been suggested to be involved in the glucotoxicity in β-cells [8,9]. An increased glycolysis due to elevated glucose converts fructose-6-phosphate and glutamine into glucosamine-6-phosphate and glutamate by a rate limiting enzyme, glutamine:fructose-6-phosphate amidotransferase (GFAT). Glucosamine-6-phospate is then metabolized to uridine diphosphate-N-acetylglucosamine (UDP-GlcNAc). Ultimately, this hexosamine pathway in the pancreatic β-cells leads to inhibition of glucose-stimulated insulin secretion (GSIS) and induction of apoptosis by decreasing the levels of glucose transporter 2 and glucokinase expressions [9,10]. These alterations in the glucose metabolism and/or extracellular fluid could contribute to production of glucotoxocity in β-cells.

In this study, to examine the direct sub-chronic effect of elevated glucose on β-cells, single β-cells were isolated from rats and cultured for 3 days with high (22.3 mM) glucose (HG), compared with control 5.6 mM glucose, to induce glucotoxicity. They were also cultured with candidate mediators and inhibitors of glucotoxicity. Following the culture, the β-cell signaling activity was assessed by measuring cytosolic Ca^2+^ concentration ([Ca^2+^]_i_) using fura-2 and its exocytotic activity was assessed using quinacrine microfluorometry in single β-cells; their responses to physiological (8.3 mM) glucose, arginine and tolbutamide were investigated. In pancreatic β-cells, a rise in glucose concentration increases intracellular ATP and closes the ATP-sensitive K^+^ (K_ATP_) channel, which depolarizes the cell membrane to open the voltage-dependent Ca^2+^ channel (VDCC), leading to an increase in [Ca^2+^]_i_ that triggers exocytosis of insulin [11,12]. Hence, the [Ca^2+^]_i_ response to glucose reflects the β-cell signaling leading to insulin secretion [13].

## RESULTS

A rise in glucose concentration from 2.8 to 8.3 mM induced an increase in [Ca^2+^]_i_ in a single β-cell following culture for 3 days under control condition with 5.6 mM glucose (Fig. 3A), and the [Ca^2+^]_i_ response occurred in 41 of 50 β-cells (82%) (Fig. 4). These β-cells also responded to administration of insulin secretagogues, a K_ATP_ channel blocker tolbutamide (300 μM) and arginine (10 mM), with increases in [Ca^2+^]_i_. In contrast, in β-cells after culture for 3 days with high (22.3 mM) glucose (HG), the [Ca^2+^]_i_ responses to 8.3 mM glucose were markedly suppressed or inhibited (Fig. 3B), and the [Ca^2+^]_i_ response occurred in 17 out of 70 β-cells (24%) (Fig. 4). The [Ca^2+^]_i_ responses to tolbutamide and arginine were also suppressed to a lesser extent than those to 8.3 mM glucose (Fig. 3B). In β-cells after culture with 16.7 mM L-glucose, an enatiomer of D-glucose, the [Ca^2+^]_i_ responses to 8.3 mM glucose, tolbutamide and arginine occurred in a similar manner to those in β-cells after control culture (Fig. 3C vs. A), and the incidence of the [Ca^2+^]_i_ response to 8.3 mM glucose was unaltered (Fig. 4).

**Figure 2:**
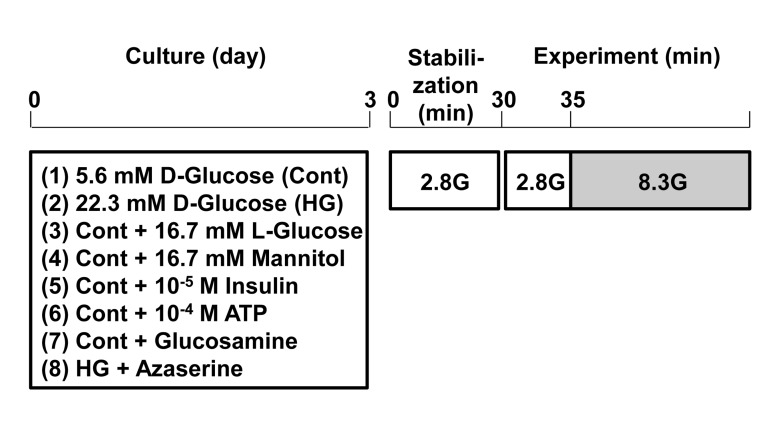
Protocol for induction of glucotoxicity and assessment of responsiveness to physiologic glucosein pancreatic β-cells. Single β-cells isolated from rats were cultured under control and test conditions for 3 days. Subsequently, single β-cells were first incubated for 30 min in HKRB containing 2.8 mM glucose for stabilization and then subjected to [Ca^2+^]_i_ measurements for functional assay.

**Figure 3:**
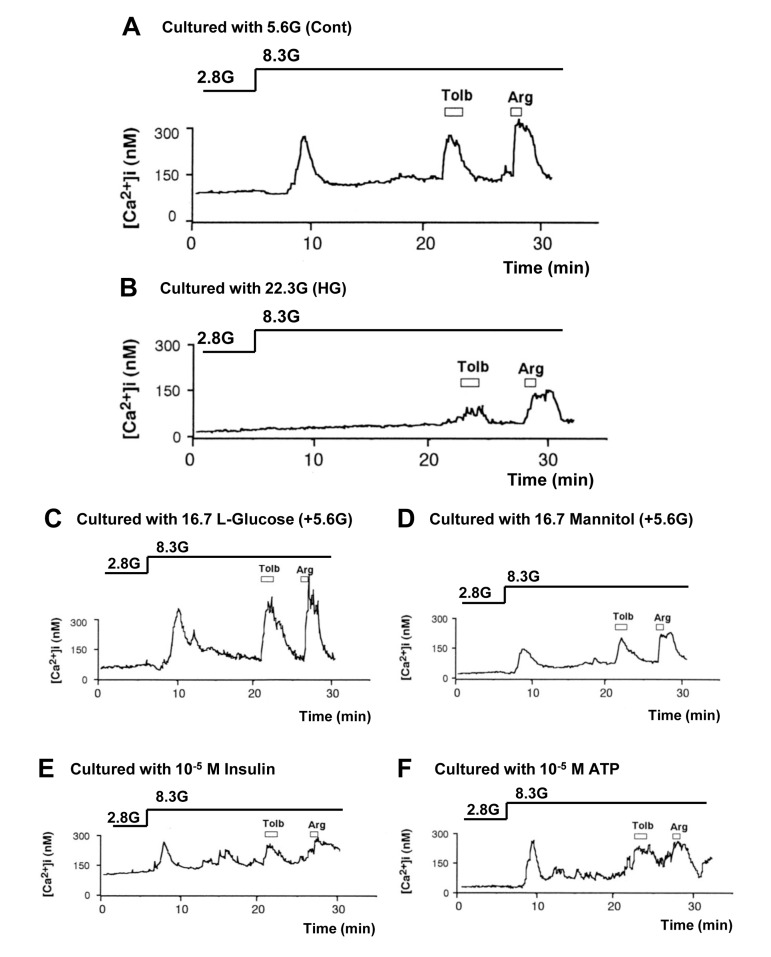
High (22.3 mM) glucose in culture impaired subsequent [Ca^2+^]_i_ responses to physiologic glucose challenge in pancreatic β-cells, while L-glucose and increases in osmolarity, insulin and ATP had no or weaker effects. **A**, [Ca^2+^]_i_ responses to 8.3 mM glucose (8.3G), 300 μM tolbutamide (Tolb), and 10 mM arginine (Arg) in single β-cells following control culture with 5.6 mM glucose. A representative of 41 cells (N=41). **B**, [Ca^2+^]_i_ responses to 8.3G, Tolb and Arg in β-cells following culture with 22.3 mM glucose. N=17. **C-F**, [Ca^2+^]_i_ responses to 8.3G, Tolb and Arg in β-cells following culture with 16.7 mM L-glucose (C; N=40), 16.7 mM mannitol (D; N=14), 10^−5^ M insulin (E; N=21), and 10^−5^ M ATP (F; N= 39) in the presence of 5.6 mM D-glucose.

**Figure 4:**
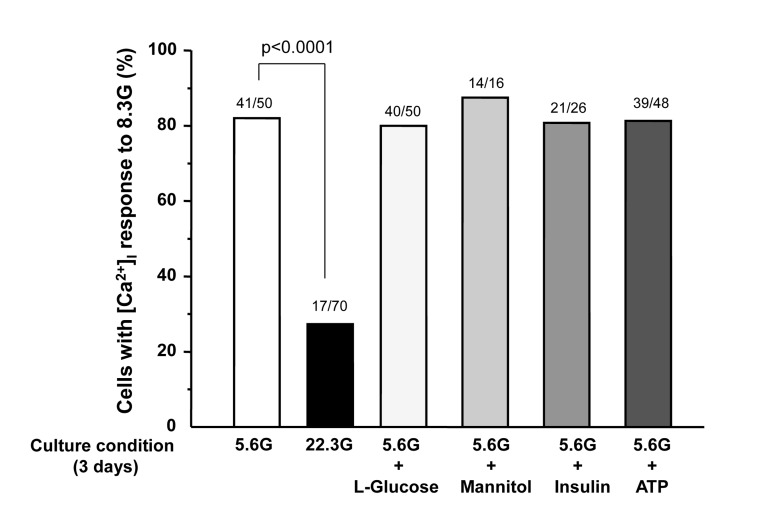
Percentage of β-cells that respond to 8.3 mM glucose after culture for 3 days in described conditions. Numbers on top of the bar indicate the number of cells that responded with [Ca^2+^]_i_ increases over that examined.

In β-cells after culture with 16.7 mM mannitol, 10^−5^ M insulin or 10^−5^ M ATP, the amplitude of the [Ca^2+^]_i_ increase in response to 8.3 mM glucose was suppressed (Fig. 3D-F), while the incidence of the [Ca^2+^]_i_ increase was unaltered (Fig. 4). The amplitude of the [Ca^2+^]_i_ response to tolbutamide and that to arginine were also suppressed. These results indicate that increases in extracellular osmolarity, insulin and ATP do not mediate the action of HG to eliminate [Ca^2+^]_i_ responses to 8.3 mM glucose. The result that the exposure to high L-glucose failed to impair β-cells suggests that the elevated metabolism of glucose is involved in the glucotoxicity in β-cells.

**Figure 5:**
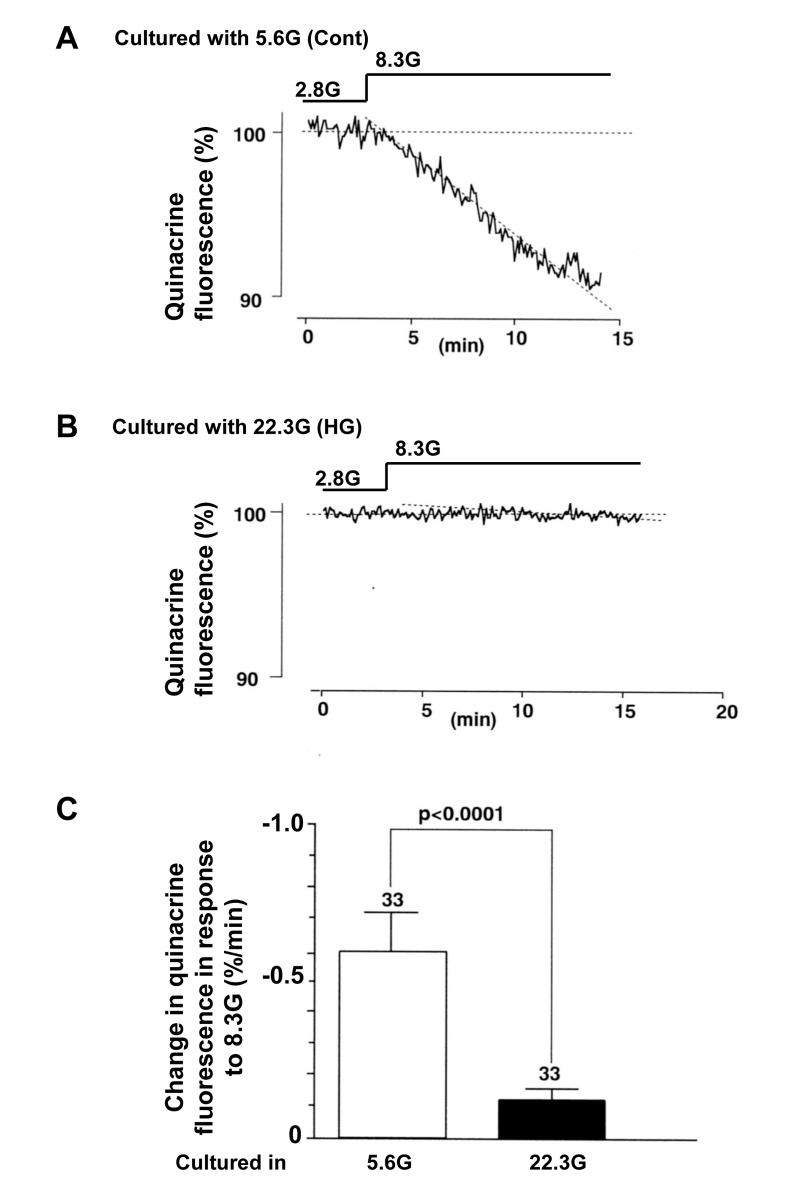
Insulin secretory response to physiologic glucose challenge assessed by decrease of quinacrine fluorescence in β-cells following culture in high (22.3 mM) glucose (**A**) and control culture in 5.6 mM glucose (**B**). **C**, Rate of quiniacrine fluorescence decrease. Numbers above the bar indicate the number of recordings.

The β-cell function related to insulin secretion was also assessed by the decrease in fluorescence of quinacrine loaded in the secretory granules of β-cells. In a single β-cell after culture with control 5.6 mM glucose for 3 days, the quinacrine fluorescence from the cell decreased substantially in response to a rise in the superfusate glucose concentration from 2.8 to 8.3 mM (Fig. 5A). In a single β-cell after culture with HG for 3 days, in contrast, the quinacrine fluorescence from the cell decreased only slightly (Fig. 5B) and the rate of decrease was significantly less than that in β-cells after control culture (Fig. 5C). These results of [Ca^2+^]_i_ and quinacrine fluorecence measurements suggest that the treatment with HG for 3 days results in impairment of the β-cell functional responses to physiological glucose stimulation.

**Figure 6:**
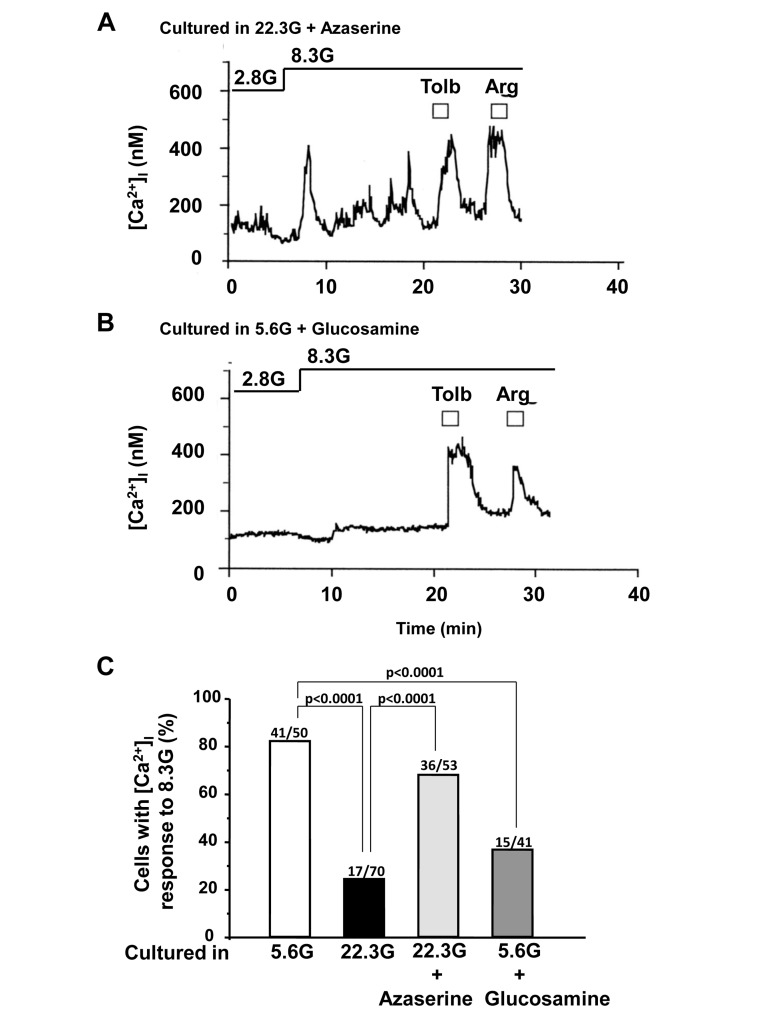
Effects of administration of azaserine and glucosamine in culture on subsequent [Ca^2+^]_i_ responses to physiologic glucose in β-cells. **A**, Azaserine added in culture prevented the high (22.3 mM) glucose-induced impairment of [Ca^2+^]_i_ responses to physiological glucose challenge. A representative of 36 cells. **B**, Glucosamine added in control culture with 5.6 mM glucose resulted in impairment of [Ca^2+^]_i_ responses to physiological glucose challenge. N=15. **C**, Incidence of [Ca^2+^]_i_ responses to 8.3 mM glucose in β-cells after culture for 3 days in described conditions. Numbers on top of bar indicate the number of cells that responded with [Ca^2+^]_i_ increases over that examined.

To examine the contribution of hexosamine pathway, azaserine (10 μM) and glucosamine (2 mM) were added to culture. The administration of azaserine, a glutamine analog that inhibits hexosamine pathway, restored [Ca^2+^]_i_ response to 8.3 mM glucose: the response occurred in 36 of 53 (68%) β-cells cultured in HG condition (22.3 mM) for 3 days (Fig. 6A,C). Conversely, administration in culture of glucosamine, which enhances the flow through the hexosamine pathway by bypassing GFAT (Fig. 1), either abolished or suppressed [Ca^2+^]_i_ responses to 8.3 mM glucose (Fig. 6B,C), while the responsiveness to tolbutamide and arginine was preserved or only partially suppressed (Fig. 6B). Thus, glucosamine mimicked HG in impairing the β-cell ability to respond to physiological glucose with [Ca^2+^]_i_ increases.

## DISCUSSION

We found that exposure of single pancreatic β-cells for 3 days to 22.3 mM glucose, the HG condition in this study, impaired their ability to respond to physiological (8.3 mM) glucose challenge, confirming previous report [14]. The novel finding of the present study is that this effect of HG is due primarily to its direct action on β-cells. The [Ca^2+^]_i_ response to a physiological glucose challenge was reduced by culture with HG. Following culture with elevated osmolarity, insulin or ATP, the incidence of [Ca^2+^]_i_ responses to 8.3 mM glucose was not altered, while their amplitude was attenuated. These conditions also attenuated the amplitude of [Ca^2+^]_i_ responses to tolbutamide and arginine. Thus, the elevated extracellular osmolarity, insulin and ATP suppressed β-cell responses to the three insulin secretagogues, a property not fitting to the glucotoxicity that refers to the reduced responsiveness preferentially to glucose [15]. Taken together, the present results indicate that the HG-induced glucotoxicity in β-cells is produced mainly via the action of glucose but not increased osmolarity, insulin and ATP, although minor contribution of the latter factors cannot be excluded.

In this study, application of azaserine, which inhibits the hexosamine pathway, prevented the action of HG in culture to impair the [Ca^2+^]_i_ response to physiological glucose challange. Conversely, administration in culture of glucosamine, which enters the hexosamine pathway by bypassing GFAT, impaired [Ca^2+^]_i_ responses to physiological glucose challange in β-cells cultured with normal 5.6 mM glucose. Although hexosamine pathway is a relatively minor branch of glycolytic pathway (about 3% of total glucose utilization) [16,17], several studies have reported that the flux through hexosamine pathway may play a major role in the development of IR and other complications accompanying diabetes [16-22]. Moreover, it has been reported that the hexosa-mine pathway serves to impair GSIS in β-cells [8,9]. These results by us and others suggest that the hexosamine pathway substantially mediates the HG action to attenuate the response to physiological glucose stimulation in β-cells. It was reported that the reduction of GSIS due to an increased flux through hexosamine pathway was due to reduced expression of key proteins or enzymes that promote insulin secretion in β-cells [9,10,22]. However, further studies are required to elucidate the precise mechanisms underlying the HG-induced impairment of the [Ca^2+^]_i_ responses to physiologic glucose challange in β-cells.

Diabetes has become one of the most pernicious and widespread health problems in the world. It is important to apply an effective treatment in the early stage of diabetes before it progresses to serious stage with various complications. Glocotoxicity in pancreatic β-cells is one of the key processes that take place from the early stage of impaired glucose tolerance and thereby initiates and accelerates type 2 diabetes. The present finding that the hexosamine pathway substantially mediates the glocotoxicity in pancreatic β-cells provides a new and effective target for the treatment of diabetes in its early stage.

## MATERIALS AND METHODS

### Preparation of single islet β-cells

Islets and isolated β-cells were prepared as previously reported [23,24]. Briefly, islets were isolated from Wister rats aged 10-16 weeks by collagenase digestion. Animals were anesthesized with intraperitoneal injection of pentobarbitone at 80 mg/kg. Islets were isolated and washed with HKRB buffer (in mmol/l: 5 CaCl_2_, 2.8 glucose, 129 NaCl, 4.7 KCl, 1.2 KH_2_PO_4_, 5 NaHCO_3_, 1.2 MgSO_4_, 10 HEPES, pH 7.4). Single β-cells were prepared from islets by treatment with Ca^2+^-free HKRB made with 0.1 mmol/l EGTA (Sigma Chemicals Co., St. Louis MO). β-cells were cultured in Eagle's MEM supplemented with 10% (v/v) fatal bovine serum, 100 mg/ml streptomycin and 100 U/ml penicillin and cultured at 37°C in a 95% air plus 5% CO_2_ atmosphere.

### Application of glucose, L-glucose, mannitol, insulin, ATP, glucosamine and azaserine

Glucose (5.6 mM, 22.3 mM), L-glucose (16.7 mM), mannitol (16.7 mM), insulin (10^−5^ M), ATP (10^−5^ M), glucosamine (2 mM) and azaserine (10 μM) (Sigma Chemicals Co., St. Louis MO) were added in culture medium for three days prior to [Ca^2+^]_i_ measurements.

### [Ca^2+^]_i_ measurements

Following culture for 3 days under control and test conditions, the cells were first incubated in HKRB containing 2.8 mM glucose for 30 min for stabilization and then subjected to the measurement of cytoplasmic Ca^2+^ concentration ([Ca^2+^]_i_) (Fig 2). [Ca^2+^]_i_ in β-cells isolated from islets were measured as previously described [13]. In brief, single β-cells were incubated with HKRB containing 3 μmol/l fura-2/AM (Dojin chemical, Kumamoto, Japan) and 2.8 mmol/l glucose for 30 min at 37°C. Following incubation, they were transferred to an open chamber mounted on the stage of fluorescence microscope. The cells were superfused with HKRB at a rate of 1 ml/min at 37°C. Fura-2 was exited alternately at 340 and 380 nm every 5 sec, and the emission fluorescence at 510 nm was detected by a cooled charge-coupled device camera, and the ratio (F340/F380) of corresponding fluorescence was calculated. Images were produced by an Argus 50 system (Hamamatsu Photonics, Hamamatsu, Japan). Ratio values were converted to [Ca^2+^]_i_ according to calibration curves. Peak values of [Ca^2+^]_i_ were used for statistical analysis. The identity of β-cells was confirmed by cell diameter and [Ca^2+^]_i_ responses to sulfonylurea tolbutamide (0.3 mM) as reported previously [13]. When changes in ratio (F340/F380) took place within 5 min after administration of reagents and their amplitudes were more than 0.4 ratio unit, they were considered to be responses.

### Assessment of exocytosis

Insulin secretion was evaluated by the loss of quinacrine fluorescence from prelabelled single β-cells upon stimulation with physiological glucose concentration. The β-cells on coverslips were incubated with 200 nM quinacrine (Sigma Chemicals Co., St. Louis MO) for 3 h at 25°C. Quinacrine microfluorometry was performed under conditions identical to those used for [Ca^2+^]_i_ measurements. The cells were excited at 340 nm every 5 sec and emission signals at 510 nm were detected with an intensified charge-coupled device (ICCD) camera. Exocytosis was assessed by the loss of the quinacrine fluorescence and expressed as a change in fluorescence intensity per minute.

### Statistical analysis

All values were expressed as mean ± SEM (n=number of observation). The statistical analysis was carried out using Tukey type multiple comparison test for rank values of variables, and χ^2^ test and Exact test by Monte Carlo.
